# Correlation of skin carotenoid levels with embryo development and pregnancy result of in vitro fertilization cycles for couples with unexplained infertility

**DOI:** 10.1002/fsn3.1615

**Published:** 2020-05-07

**Authors:** Wen‐Jung Chen, Shu‐Ling Tzeng, En‐Hui Cheng, Hui‐Mei Tsao, Chun‐Chia Huang, Sung‐Lang Chen, Maw‐Sheng Lee, Tsung‐Hsien Lee

**Affiliations:** ^1^ Institute of Medicine Chung Shan Medical University Taichung Taiwan; ^2^ Department of Urology Chung Shan Medical University Hospital Taichung Taiwan; ^3^ School of Medicine Chung Shan Medical University Taichung Taiwan; ^4^ Division of Infertility Clinic Lee Women's Hospital Taichung Taiwan; ^5^ Department of Obstetrics and Gynecology Chung Shan Medical University Hospital Taichung Taiwan

**Keywords:** antioxidants, carotenoid, embryo quality, unexplained infertility

## Abstract

Oxidative stress‐related DNA damage is a significant pathology for male subfertility and unexplained infertility (UI). Antioxidant supplement by food or nutrition may benefit sperm function of UI couples. However, the role of antioxidant status on fertilization outcome and embryo development for UI couples is not clear. A total of 63 semen samples from UI couples undergoing in vitro fertilization (IVF) treatment (26 pregnant cycles and 37 nonpregnant cycles) were recruited for this prospective observational study. The reactive oxygen species (ROS) levels of sperm cells are detected by a chemiluminescence assay. Total antioxidant capacity (TAC) of seminal plasma is evaluated according to an antioxidant assay kit. The skin carotenoid status in the male partners of UI couples is measured by resonance Raman spectroscopy to determine the antioxidant potential from dietary supplement. The skin carotenoid status (23,115 ± 6,831 vs. 19,432 ± 5,242 Raman intensity, *p* = .0329 by Mann–Whitney *U* test) and day 3 good embryo rates (49.6 ± 27.1% vs. 26.8 ± 23.1%, *p* = .002 by Mann–Whitney *U* test) are higher in pregnant cycles compared to those in nonpregnant cycles. The local antioxidant capacity (seminal TAC) is closely correlated with fertilization rates (*r* = .35, *p* = .005). In contrast, skin carotenoid status is intimately associated with good embryo rates in IVF cycles (*r* = .34, *p* = .007). In conclusion, the skin carotenoid status of male partners of UI couples may benefit embryo development and the subsequent pregnancy outcome of IVF treatment. Further investigation about the effect and mechanism of nutritional supplement on embryo development in IVF cycles for UI couples is deserved.

## Graphical abstract

The skin carotenoid levels of male partners are positively correlated with the good embryo rates and pregnancy outcome in in vitro fertilization (IVF) cycles for couples with unexplained infertility. Whether food or nutrition supplement could improve embryo quality in ART cycles deserves further investigation.

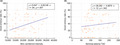

## 1 INTRODUCTION

Oxidative stress‐related injury to sperm cells is a significant cause leading to male subfertility and unexplained infertility (Agarwal, Prabakaran, & Allamaneni, 2006; Bonanno et al., 2016; Said et al., 2004; Tremellen, 2008). Reports indicated a reduction of antioxidant potential in seminal fluid of the subfertile men compared to that of the fertile men (Mostafa et al., 2006; Song, Norkus, & Lewis, 2006). An elevated level of reactive oxygen species (ROS) in seminal fluid results in impairment of sperm DNA integrity and inhibition of sperm cell activity (Moustafa et al., 2004). Furthermore, oxidative stress in the semen was identified from normozoospermic men undergoing infertility survey (Pasqualotto et al., 2001). These findings suggest that evaluating the levels of antioxidant capacity, ROS, and DNA injury in the semen may be an important work‐up for infertile couples in addition to basic semen analysis to determine the fertilization capability of spermatozoa.

During sperm preparation for in vitro fertilization (IVF) cycles, the spermatozoa are prone to exposure to a transiently high level of ROS due to removal of seminal plasma after in vitro manipulation, such as density gradient centrifugation (DGC; Aitken, De Iuliis, Finnie, Hedges, & McLachlan, 2010; Aitken et al., 2014). Furthermore, antioxidants have been added into the culture media for sperm preparation/wash to decrease the oxidative stress and to benefit sperm activity in intracytoplasmic sperm injection (ICSI) cycles (Busetto et al., 2018; Chi et al., 2008). Nonetheless, several reports indicated that ROS is positive for capacitation‐associated modification (Aitken, Harkiss, Knox, Paterson, & Irvine, 1998; de Lamirande & Lamothe, 2009; O'Flaherty, de Lamirande, & Gagnon, 2005). The elevated ROS levels in washed spermatozoa could relate to sperm capacitation. The level and location of tyrosine phosphorylation pattern are directly influenced by sperm ROS content, which allow the spermatozoa to undergo acrosome reaction (Dona et al., 2011). Taking together, the ROS content in neat semen or washed spermatozoa plays a dual role in the progression of capacitation and fertilization (Aitken, Gordon, et al., 1998). The effect of the antioxidant capacity in semen on fertilization and embryo development of UI couples undergoing IVF cycles deserves further investigation.

The reduction potential of semen depends on antioxidant contents in the seminal plasma. It has been reported that dietary antioxidants, specifically carotenoid, are closely related to semen quality in a young healthy population (Zareba et al., 2013). A dietary pattern abundant in vegetables, fruits, seafood, and whole grains (Mediterranean diet) is related to improving sperm function for the couples undergoing fertility survey (Karayiannis et al., 2017; Salas‐Huetos, James, Aston, Jenkins, & Carrell, 2019) or healthy men (Cutillas‐Tolin et al., 2015). Carotenoids are plant pigments resulting in orange, red, and yellow coloring and are categorized as antioxidants. The carotenoid contents in the body/tissue are currently the best biomarkers for vegetable and fruit intake (Mayne et al., 2010; Scarmo et al., 2012). The performance of this antioxidant status from food supplement for sperm function in IVF cycles has rarely been investigated.

To evaluate the oxidative–reductive potential in the ejaculate, we choose the total antioxidant capacity (TAC) of semen, ROS levels of neat semen, and washed spermatozoa as biomarkers to represent the local reductive–oxidative status. The skin carotenoid intensity is selected as the marker of systemic antioxidant status related to dietary supplement. The object of this study is to elucidate the connection among systemic/local antioxidant potential and sperm function in IVF cycles for normozoospermic men without female infertility factors (UI couples). The relevance of the antioxidant status in the body and semen to the fertilization, in vitro embryo development, and pregnancy outcome in IVF cycles was explored. Data in the present study might provide a possible new approach for the evaluation and management with nutrition supplement for male partners of UI couples.

## 2 MATERIALS AND METHODS

### 2.1 Patient selection and semen sample collection

The approval of the Institution Review Boards of Chung Shan Medical University Hospital, Taichung, Taiwan (CS2‐17008), was obtained for all the study procedures. Only infertile couples with UI were recruited for the present study. Unexplained infertility is a diagnosis of exclusion after detailed infertility work‐up, including ovarian hormone tests, hysterosalpingography or laparoscopy, and basic semen analysis, that has failed to reveal any abnormalities (Lee et al., 2010). A total of 63 male partners of UI couples during semen analysis and sperm preparation for the IVF cycles provided the semen samples. The participating couples recruited from July 2014 to June 2016 signed the informed consents.

Subsequent to a period of 3–5 days of sexual abstinence, the semen analysis was executed based upon the 5th edition of World Health Organization (WHO) guidelines (WHO, 2010). All semen samples feature normal semen analysis (sperm concentration >15 × 10^6^/ml, total motility >40%, and strict morphology >4%) and Endtz test <1.0 × 10^6^/ml. Endtz test can be used to distinguish germinal cells from polymorphonuclear granulocyte (PMNs) and both are round cells. If the result of Endtz test is <1.0 × 10^6^/ml, the semen sample is thought to be free of leukospermia, in which the ROS level would be influenced by PMNs.

All the spermatozoa were prepared with modified HTF (mHTF) media and DGC method after basic semen analysis and in the IVF cycles, which is the same as described in our previous studies (Lee et al., 2010; Shih et al., 2016). The measurement of prewashed ROS, postwashed ROS, and preparation for TAC was performed on the day 2 to day 5 of the ovarian stimulation cycles of the female partners. The skin carotenoid status was detected on the day of oocyte retrieval for the UI couples. One aliquot of liquefied neat semen was used for analysis of ROS evaluation. The liquefied semen was mixed with mHTF media accompanied with 10% inactivated human serum (Baxter Healthcare Corporation, Baxter Bioscience). The motile sperm selection by DGC were using the PureSperm (Nicadon) with two layers of 1 ml 40% and 1 ml 80% PureSperm at 300 g for 20 min. The supernatant seminal plasma was then carefully retrieved and frozen at −80°C until TAC examination. The sperm pellet was transported to a new tube and was resuspended in 1 ml mHTF media. Then, ROS levels of spermatozoa after washing were measured.

### 2.2 Measurement of oxidative injury and DNA damage in spermatozoa

The residual semen samples obtained after basic semen analysis were collected for these measurements. We use the Calbiochem^®^ OxyDNA Kit (Merck KGaA) to evaluate the oxidative DNA injury in spermatozoa. The method is the same as that described in our previous report (Shih et al., 2016). One aliquot of semen with 3 x 10^6^ spermatozoa was pelleted and washed with phosphate‐buffered saline (PBS), then fixed, permeabilized sperm cells with 70% ethanol, and kept at −20°C for 1 hr. Then, we put in 100 µl 1× FITC‐Conjugate to the cell pellet and incubate for 60 min. A flow cytometer was used to read fluorescence at a barrier filter of 515 nm and an excitation wavelength of 495 nm.

Terminal deoxynucleotidyl transferase‐mediated deoxyuridine triphosphate‐biotin nick‐end labeling (TUNEL) assay (Boehringer Mannheim, Mannheim, Germany) was used to measure DNA fragmentation as in our previous reports (Lee et al., 2010; Shih et al., 2016). Briefly, the samples of sperm cells were washed in PBS, followed by centrifugation at 200 *g* for sperm collection. The spermatozoa were manipulated with 0.1% Triton X‐100 (Sigma) and then incubated with TUNEL mixture at 37°C for 60 min in darkness. The samples were washed with PBS three times and then analyzed in a FACS.

### 2.3 Measurement of ROS levels

A chemiluminescence assay using the probe luminol (5‐amino‐2, 3‐dihydro‐1,4‐phthalazinedione; Sigma) was performed to measure the ROS levels, which is described in detail in our previous reports (Lee et al., 2012; Shih et al., 2016). A luminometer (FlexStation 3 Benchtop MultiMode Microplate Reader; Molecular Devices, LLC) was used to scan each semen or sperm sample. All the samples were measured in duplicate. The ROS values were expressed as relative light units (RLU).

### 2.4 Measurement of TAC in seminal plasma

Seminal plasma TAC measurement was operated according to the antioxidant assay kit (Cayman Chemical) that is similar to previous reports (Mahfouz, Sharma, Sharma, Sabanegh, & Agarwal, 2009; Miller & Rice‐Evans, 1997). The assay measures the ability of total antioxidants in the seminal plasma to inhibit the oxidation of the 2,20‐Azino‐di‐[3‐ethylbenzthiazoline sulphonate] (ABTS) to ABTSt. The antioxidant capacity to inhibit ABTS oxidation in the sample was compared to that of a water‐soluble tocopherol analogue, Trolox. ELx800 Absorbance Microplate Reader (BioTek Instruments, Inc.) was used to monitor absorbance at 750 nm.

### 2.5 Assessment of skin carotenoid status

The skin carotenoid status was evaluated by resonance Raman spectroscopy (RRS). RRS is a less expensive, noninvasive, and rapid way to assess carotenoid status for estimating vegetable and fruit intake (Mayne et al., 2010; Scarmo et al., 2012). It has been proved that RRS is a valid way to measure carotenoid status in healthy adult population (Scarmo et al., 2010).

Resonance Raman spectroscopy instrumentation has been reported in detail elsewhere (Ermakov, Ermakova, McClane, & Gellermann, 2001; Scarmo et al., 2012), and we used it with minor modifications in the present study. In short, the instrument has a portable, compact, continuous‐wave solid‐state laser working at a wavelength of 488 nm to shine blue visible light onto a tissue of interest (i.e., the palm of patients). The palm of each patient was checked twice at an exposure time of 30 s. The examination was completed on the day of ovum pick‐up for female partners of UI couples. The total skin carotenoid status of each patient was determined by the mean RRS values, quantified as skin Raman intensity.

### 2.6 The protocol for in vitro fertilization and embryo quality evaluation

The ovarian stimulation procedures employed in the present study were the long protocol for the use of gonadotropin‐releasing hormone agonist (GnRHa), which was published elsewhere (Lee et al., 2012; Shih et al., 2014). In brief, women were injected with the GnRHa, leuprolide acetate, to achieve pituitary desensitization. The women were subsequently treated with recombinant follicular stimulation hormone. When dominant follicles reached 18 mm in diameter, recombinant human chorionic gonadotropin (hCG) was injected. The ovum pick‐up procedure was done 36 hr after hCG administration. The retrieved oocytes were placed in a triple‐gas‐phase (5% CO_2_, 5% O_2_, and 90% N_2_) incubator at 37°C within a mHTF medium.

The DGC technique was used to select motile spermatozoa. The oocytes were inseminated with an average of 10,000 motile spermatozoa for one oocyte in a mHTF media. In vitro development of embryos at the eight‐cell stage (69–70 hr) was checked. The embryos at day 3 were classified according to the criteria as our previous report (Lee et al., 2012). In brief, the embryos with 6–10 equally sized blastomeres and less than 15% fragmentation were classified as good quality. Those embryos with 15%–50% fragmentation and unequally or equally sized blastomeres were classified as fair quality. The embryos were defined as poor quality if the fragmentation was above 50%. Only good or fair quality embryos were selected for transfer. For all patients in the present study, one to three embryos were transferred in the present study. The number of transferred embryos was determined by female age and embryo quality. The good embryo rates on day 3 were computed as follows:

$$\text{rate}\;\left(\%\right)=\left(\text{number}\;\text{of}\;\text{good}\;\text{embryos/total}\;\text{zygotes}\right)\times 100$$

### 2.7 Statistical analysis

Wilcoxon signed‐rank test was used for self‐comparison of sperm ROS levels, percentage of spermatozoa with oxidative DNA injury (8OH‐DG positive), and DNA fragmentation (TUNEL positive) between pre‐ and post‐DGC preparation of the semen samples for basic semen analysis. Mann–Whitney *U* test was utilized to evaluate the relevance of the systemic and local antioxidant status to fertilization rates and good embryo rates in ART cycles. Linear regression models are utilized to evaluate the independent effect of systemic or local antioxidant status upon fertilization rates and good embryo rates in IVF cycles for UI couples. A confidence level of *p* < .05 was considered as statistically significant. The data analyzed in the present study are supplemented as the file Data S1. 

## 3 RESULTS

Sixty‐three UI couples undertaking IVF treatment participated in this prospective observational study. Table 1 shows the baseline and biomarker data of the UI couples. The female age, female BMI, pregnancy history (gravida, para, abortion), female AMH levels, previous IVF cycles, duration of infertility, male age, male BMI, and basic semen analysis data were not significantly different between pregnant (*n* = 26) and nonpregnant (*n* = 37) couples. Interestingly, skin carotenoid status of male partners was significantly different between pregnant and nonpregnant couples (23,115 ± 6,831 vs. 19,432 ± 5,242 Raman intensity, *p* = .0329 by Mann–Whitney *U* test; Table 1).

**Table 1:** Demographic data and IVF treatment results for the couples with unexplained infertility (*n* = 63)

IVF cycles (*N* = 63)	Pregnancy (*n* = 26)	Nonpregnancy (*n* = 37)	*p* value
Female age (years)	34.6 ± 5.2	34.6 ± 5.3	.8996
Female BMI (kg/m^2^)	21.6 ± 3.5	21.7 ± 2.9	.6400
Gravida	0.5 ± 0.6	0.3 ± 0.5	.0676
Para	0.1 ± 0.3	0.1 ± 0.3	.9285
Abortion	0.3 ± 0.6	0.1 ± 0.3	.1550
Female AMH (ng/ml)	4.45 ± 2.43	3.48 ± 2.67	.0564
Duration of infertility (years)	3.50 ± 2.59	4.23 ± 3.05	.2650
Previous IVF cycles	0.1 ± 0.3	0.1 ± 0.3	.6505
Male age (years)	37.5 ± 5.2	36.0 ± 5.5	.1399
Male BMI (kg/m^2^)	28.8 ± 4.6	28.0 ± 3.9	.7166
Basic semen analysis			
Volume (ml)	2.9 ± 1.3	3.3 ± 1.5	.4507
Concentration (10^6^/ml)	126.7 ± 62.2	108.6 ± 78.1	.1146
Motility (%)	70.5 ± 23.7	69.7 ± 24.4	.9221
Morphology (%)	14.2 ± 7.6	11.5 ± 6.7	.1773
Oocyte number	8.9 ± 5.0	7.6 ± 6.6	.1026
Zygote number	7.2 ± 4.3	6.2 ± 6.0	.0542
Fertilization rates (%)	81.4 ± 12.3	81.2 ± 19.3	.5631
Good embryo rates (%)	49.6 ± 27.1	26.8 ± 23.6	.002
Embryo transfer number	2.3 ± 0.6	2.2 ± 0.9	.816
Number of transferred good embryos	2.0 ± 0.9	1.4 ± 1.2	.0314
8‐OH DG (%)	48.3 ± 25.6	48.5 ± 22.7	.7587
TUNEL (%)	8.3 ± 3.0	7.6 ± 2.7	.4894
Neat semen ROS (RLU)	10,438 ± 2,273	10,004 ± 2,531	.4593
Seminal plasma TAC (μmoles of Trolox equivalents)	131.4 ± 51.0	109.2 ± 47.1	.0604
Skin carotenoid status (Raman intensity)	23,115 ± 6,831	19,432 ± 5,242	.0329

Note

The percentage of spermatozoa with oxidative DNA injury (8‐OH DG) and DNA fragmentation (TUNEL) is presented. TAC and ROS denote total antioxidant capacity and reactive oxygen species, respectively. Relative light unit (RLU) is the unit for ROS measurement. The data are presented as the mean ± *SD*. *p* value is determined by Mann–Whitney *U* test.

The retrieved oocyte numbers, fertilization rates, good embryo rates, number of transferred good embryos, and embryo transfer numbers after IVF treatment are also shown in Table 1. The retrieved oocyte numbers, fertilization rates, and embryo transfer numbers are not significantly different between pregnant and nonpregnant IVF cycles. Nonetheless, the day 3 good embryo rates are higher in pregnant cycles compared to those in nonpregnant cycles (49.6 ± 27.1% vs. 26.8 ± 23.1%, *p* = .002 by Mann–Whitney *U* test). Subsequently, the number of transferred good embryos is also higher in pregnant cycles than that in nonpregnant ones (2.0 ± 0.9 vs. 1.4 ± 1.2, *p* = .0314 by Mann–Whitney *U* test; Table 1).

**Figure 1:**
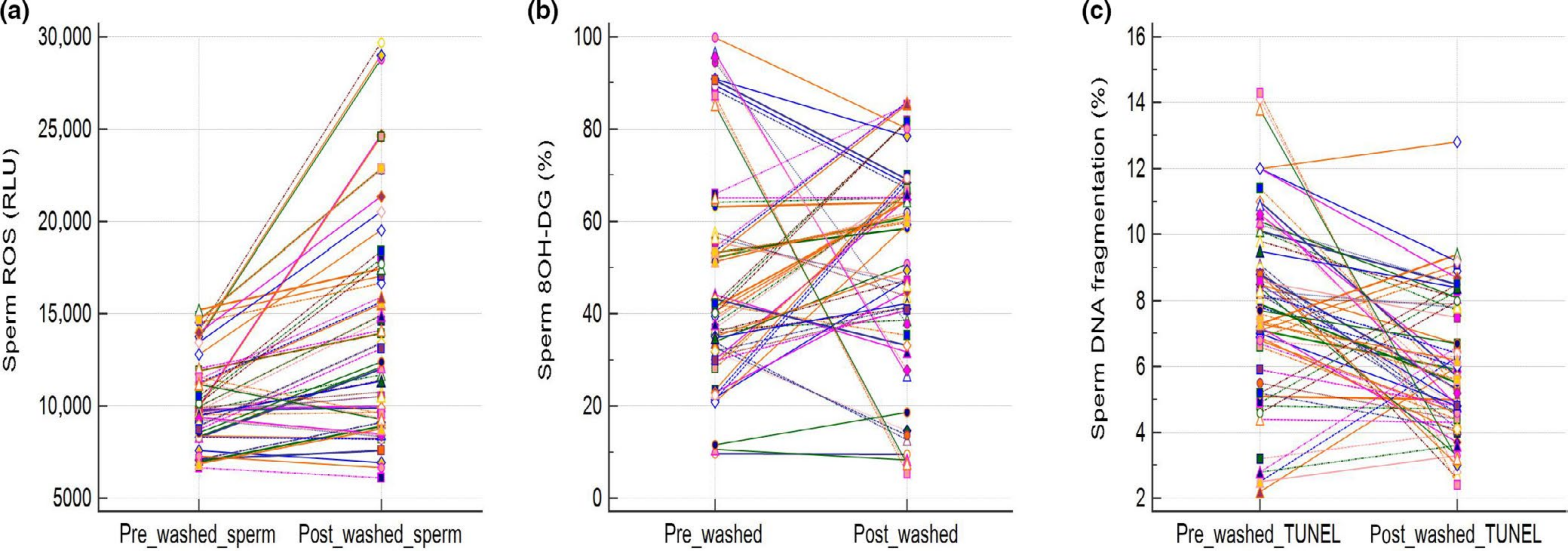
Reactive oxygen species (ROS, a), oxidative DNA injury (8‐OH DG, b), and DNA fragmentation (TUNEL, c) of spermatozoa prior to and after sperm preparation with density gradient centrifugation (DGC) method in IVF cycles. The comparison by Wilcoxon signed‐rank test revealed (a) *p* < .001, (b) *p* = .064, (c) *p* < .001, respectively

To evaluate the ROS‐induced oxidative DNA injury by the DGC preparation method, we measured ROS levels, TUNEL, and 8‐OH DG levels for pre‐ and postwashed spermatozoa by the residual semen samples collected after basic semen analysis for UI couples. The washed spermatozoa featured higher levels of ROS than neat semen (median [25 percentile–75 percentile] 12,114 [9,107–17,364] RLU vs. 9,781 [8,361–11,531] RLU, *p* < .001 by Wilcoxon signed‐rank test; Figure 1a). The 8‐OH DG did not change significantly after the DGC preparation (41.5 [32.1–57.3]% vs. 60.0 [39.2–67.0]%, *p* = .064 by Wilcoxon signed‐rank test; Figure 1b), while the TUNEL assay indicated a significant reduction after DGC preparation (7.9 [6.6–9.7]% vs. 5.6 [4.4–7.8]%, *p* < .001 by Wilcoxon signed‐rank test; Figure 1c).

The change of ROS levels, TUNEL, and 8‐OH DG levels for pre‐ and postwashed spermatozoa from prewashed to postwashed spermatozoa is compared in Table 2. Similar change was identified between pregnancy and nonpregnancy cycles.

**Table 2:** The relationship of the reactive oxygen species (ROS) levels, oxidative DNA injury (8‐OH DG), and DNA fragmentation (TUNEL) for pre‐ and postwashed spermatozoa from the male partner of couples with unexplained infertility undergoing IVF cycles

	Pregnancy (*n* = 26)		Nonpregnancy (*n* = 37)	
	Prewashed	Postwashed	Prewashed	Postwashed
Sperm ROS (RLU)	9,912 (8,893–11,865)*	12,747 (9,242–19,750)*	9,763 (7,934–11,595)**	12,020 (8,835–16,152)**
8‐OH DG	41.3 (29.4–56.8)	47.3 (37.6–66.2)	41.5 (33.9–63.6)	64.4 (38.3–67.6)
TUNEL	8.2 (6.4–10.7)^†^	6.3 (4.5–7.9)^†^	7.8 (6.1–9.5)^††^	5.4 (4.4–7.2)^††^

Note

RLU denoted relative light unit. The data are presented as median (25 percentile–75 percentile).

* *p* < .001, ^**^ *p* < .001, ^†^ *p* = .005, and ^††^ *p* = .001 by Wilcoxon signed‐rank test.

**Figure 2:**
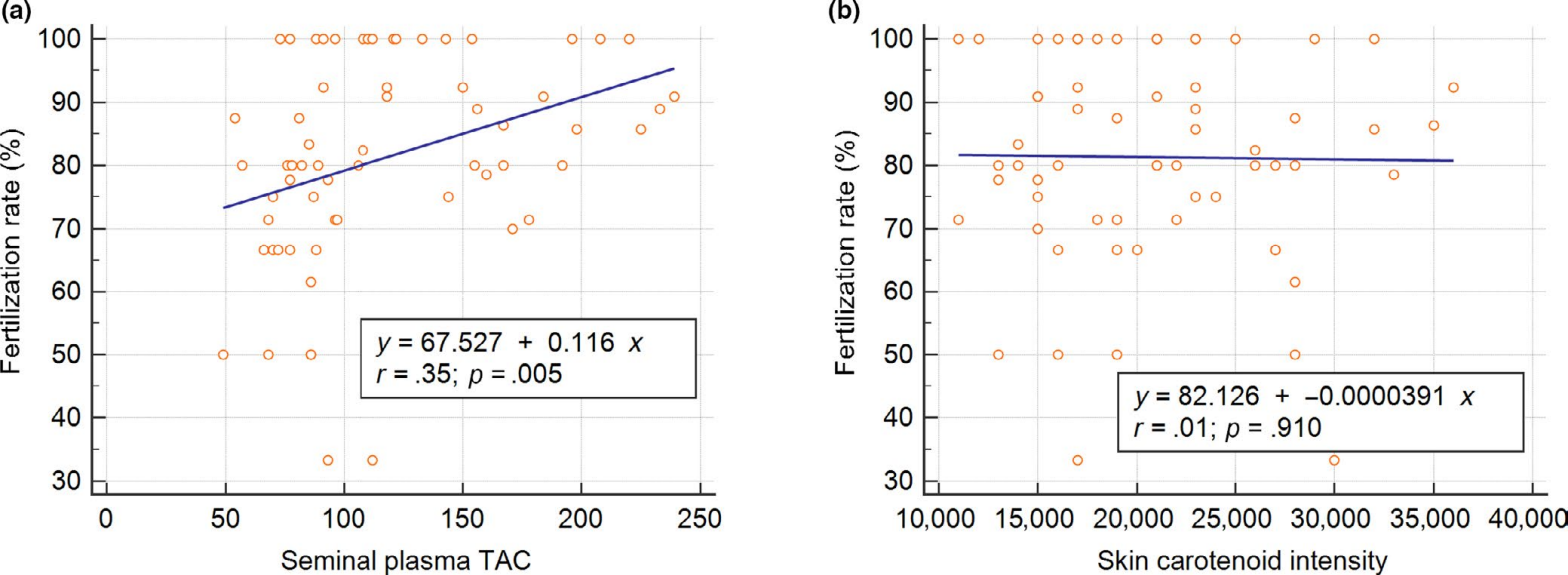
The relevance of antioxidant status in the male partners of couples with unexplained infertility to the fertilization rates in IVF cycles. (a) Carotenoid status of skin (Raman intensity). (b) Total antioxidant capacity (TAC) of seminal plasma

**Figure 3:**
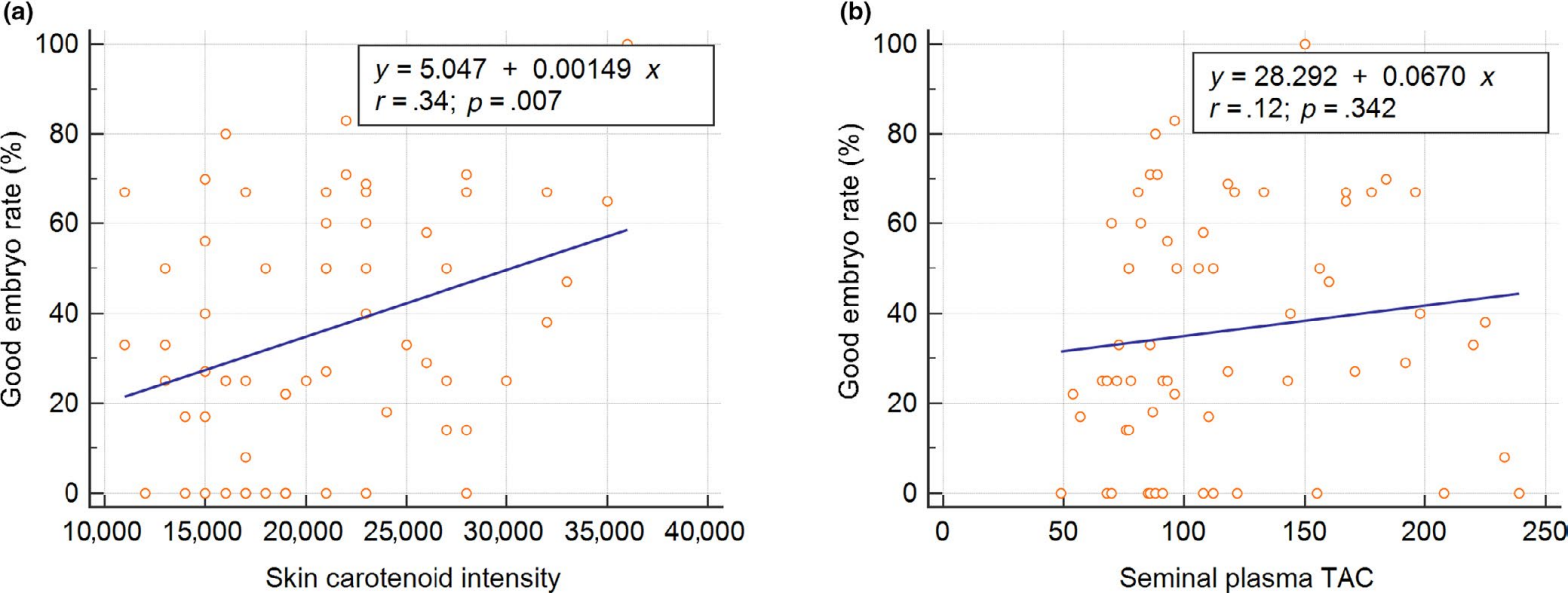
The relevance of antioxidant status in the male partners of couples with unexplained infertility to day 3 good embryos rates in IVF cycles. (a) Carotenoid status of skin (Raman intensity). (b) Total antioxidant capacity (TAC) of seminal plasma

We use linear regression analysis to explore the relevance of the antioxidant status of male partners to the in vitro fertilization rates and good embryo rates in IVF cycles. We found that TAC levels of seminal plasma (correlation coefficient, *r* = .35, *p* = .005) but not skin carotenoid status are significantly correlated with normal fertilization rates (Figure 2a,b). In contrast, skin carotenoid status (*r* = .34, *p* = .007) but not seminal plasma TAC is related to day 3 good embryo rates (Figure 3a,b). Nonetheless, the ROS levels of washed spermatozoa did not correlate with good embryo rates in ART cycles for UI couples.

The pregnancy and live birth rates are 41.3% (26/63) and 38.1% (24/63), respectively, in the present study. Six of the 26 pregnancy are twins and 20 are singleton. The multiple pregnancy rate is 23.1% (6/26). Nonetheless, a miscarriage rate of 7.7% (2/26) is noted due to two of the pregnancy results in miscarriage (abortion before a gestational age of 20 weeks).

To further clarify the effect of skin carotenoid intensity on the outcome of ART treatment for UI couples, we further divided the patients into low (*n* = 31) and high (*n* = 32) groups for further comparison (Table 3). The basic characteristics of the UI couples (male age, male BMI, basic semen analysis results, female age, female BMI, female serum AMH levels) between the two groups do not show significant difference. In addition, the seminal plasma TAC did not correlate with skin carotenoid intensity. As expected, the male partners of UI couples with high skin carotenoid intensity demonstrated higher rates of good embryos (46.8 ± 26.1 vs. 25.3 ± 24.4, *p* = .0019 by Mann–Whitney *U* test) and live birth (53.1% [17/32] vs. 22.6% [7/31], *p* = .0133 by chi‐square test) compared with those of low skin carotenoid intensity.

**Table 3:** IVF treatment results for the couples with unexplained infertility (*n* = 63) related to the skin Raman intensity

	Low skin Raman intensity (*n* = 31)	High skin Raman intensity (*n* = 32)	*p* value
Skin carotenoid status (Raman intensity)	15,839 ± 2,451	25,906 ± 4,350	<.0001
Seminal plasma TAC (μmoles of Trolox equivalents)	112.5 ± 51.6	124.0 ± 47.6	.2481
Male age (years)	36.5 ± 5.6	36.8 ± 5.2	.6394
Male BMI (kg/m^2^)	28.7 ± 3.8	28.0 ± 4.5	.3222
Basic semen analysis			
Concentration (M/ml)	114.7 ± 68.7	117.5 ± 76.1	.9507
Motility (%)	72.3 ± 24.6	67.8 ± 23.5	.3640
Morphology (%)	13.8 ± 7.1	11.5 ± 7.1	.1502
Sperm DNA fragmentation (TUNEL)	7.44 ± 2.91	8.31 ± 2.73	.2289
Female age (years)	34.1 ± 5.5	35.1 ± 5.0	.3923
Female BMI (kg/m^2^)	21.9 ± 3.1	21.4 ± 3.2	.2453
Female AMH (ng/ml)	3.67 ± 2.08	4.08 ± 3.04	.9120
Oocyte number	11.2 ± 7.1	12.3 ± 8.5	.7567
Fertilization rates (%)	79.7 ± 17.8	82.8 ± 15.6	.4787
Good embryo rates (%)	25.3 ± 24.4	46.8 ± 26.1	.0019
Embryo transfer number	2.2 ± 0.8	2.2 ± 0.8	.8943
Number of transferred good embryos	1.4 ± 1.1	1.9 ± 1.0	.0505
Pregnancy rate (%)	29.0 (9/31)	53.1 (17/32)	.0541
Live birth rate (%)	22.6 (7/31)	53.1 (17/32)	.0133

Note

The couples were divided into two groups by the level of skin carotenoid Raman intensity: low (*n* = 31) versus high (*n* = 32). The data are presented as the mean ± *SD* or percentage. *p* value is determined by Mann–Whitney *U* test or chi‐square test.

## 4 DISCUSSION

The results of the present study indicate that washed spermatozoa after DGC are associated with significantly transient elevated ROS levels but not significantly increased oxidative DNA injury to the spermatozoa in the male partners of UI couples. By contrast, the apoptotic levels (TUNEL assay) of washed spermatozoa significantly decrease compared to those of neat semen. The present study is in line with previous reports (Aitken et al., 2014; Lee et al., 2010) that DGC method could decrease the percentage of sperm cells with apoptotic markers. In the previous report, we also suggested that sperm cell function of UI couples could be improved after removal of the apoptotic sperm cells (Lee et al., 2010). The results of present study are in consistent with our aforementioned report for normozoospermic men that the transient elevation of ROS levels was noted in sperm preparation media (Shih et al., 2016), which may be related to sperm capacitation instead of oxidative DNA damage.

Male partners of UI couples were reported to have reduced levels of seminal plasma TAC and increased oxidative stress compared to fertile control (Moustafa et al., 2004). The present study further explores the correlation between antioxidant status and fertilization rate in IVF cycles. Our results indicated that the local (seminal TAC levels) instead of systemic (skin carotenoid intensity) antioxidant status in male partners of UI couples intimately correlated with the fertilization rates in IVF cycles. The seminal plasma TAC may have to compensate or against the transiently elevated sperm ROS levels to maintain the fertilization potential of spermatozoa in UI couples. If the antioxidant capacity was overwhelmed by washed sperm ROS, the oxidative stress might result in low fertilization rates in IVF cycles. Consequently, we suggest that the antioxidant supplementation to sperm culture media may also benefit fertilization results in IVF cycles for UI couples.

In the present study, skin carotenoid status within the body but not TAC in the seminal plasma is related to good embryo rates in IVF cycles. In addition, both the skin carotenoid status and good embryo rates correlated with pregnancy results in IVF cycles for UI couples. The skin carotenoid status is a marker for fruit and vegetable intake (Mayne et al., 2010; Scarmo et al., 2012); in other words, high skin carotenoid levels indicate the diet style is closely adhered to the Mediterranean diet or a dietary pattern rich of antioxidants for the male partners of UI couples. The amount of vegetable and fruit intake (Braga et al., 2015) or “Mediterranean” dietary supplement (Kermack et al., 2020) in the female partners of infertile couples undergoing IVF cycles is correlated with the embryo quality at cleavage stage. The results of the present study further suggested that, in addition to the female partners, the amount of fruit and vegetable intake in the male partners of UI couples might also contribute to the embryo quality and pregnancy outcome in IVF treatment.

The sperm parameters are better in those male partners of infertile couples with a dietary pattern rich in antioxidants, including carotenoid. Higher sperm motility is correlated with high carotenoid intake in young health men (Zareba et al., 2013). In addition, vitamin A supplementation could prevent nuclear damage (less fragmented DNA) in round spermatid in an in vitro sperm maturation model (Dumont et al., 2016). Although the sperm DNA fragmentation is not significantly different between the high and low skin carotenoid groups, the good embryo rates are intimately correlated with the skin carotenoid status of male partners of UI couples. The results supported the idea that elevation of systemic antioxidant potential by food or nutrition supplement may benefit embryo development in IVF cycles for UI couples, which is similar to those reports for infertile couples with male factor (Busetto et al., 2018; Showell et al., 2014; Tremellen, 2008). Taking together, we suggest that the insufficient antioxidants or nutrition supplement during spermatogenesis may result in occult spermatozoa damage for male partners of UI couples and subsequently impair the embryo development. Therefore, the skin carotenoid status but not seminal TAC is positively correlated with good embryo rates in IVF cycles for UI couples.

It has been reported that the infertile couples taking preconception diet intimately follow the Mediterranean pattern is associated with an elevated chance of pregnancy after IVF treatment (Karayiannis, Kontogianni, Mendorou, Mastrominas, & Yiannakouris, 2018; Vujkovic et al., 2010). High follicular folate and vitamin B6 were noted in the Mediterranean diet type group (Vujkovic et al., 2010). The embryo quality did not correlate with the Mediterranean diet score in the prospective cohort study (Karayiannis et al., 2018), but embryo quality by time‐lapse monitoring was improved in a randomized clinical trial (RCT) for couples taking Mediterranean‐like dietary pattern (Kermack et al., 2020). The present study did not use subjective questionnaire for survey; instead, we objectively measured the skin carotenoid levels at the follicular phase of female partners in the stimulation cycles. This may explain why our results by skin carotenoid status for fruit and vegetable intake are in line with those in the RCT with Mediterranean‐like dietary pattern. The limitation of the present study is that we only measured skin carotenoid status for male partners, but not female partners, of UI couples. The couples may take similar diet style in daily life. Consequently, the female partners of UI couples may have similar skin carotenoid status. Recent reports demonstrated that greater adherence to Mediterranean diet of young nonobese women is related to higher good embryo rates (Braga et al., 2015) and pregnancy rates in IVF cycles (Karayiannis et al., 2018). Due to the intimate correlation between skin carotenoid status of the male partners of UI couples on the embryo quality and pregnancy results in the present study, we may propose that the skin carotenoid status of female partners could be closely similar with that of male partners of UI couples. Since the potential predictors of embryo quality, including female age, duration of infertility, and AMH levels, are not different in the present study, the skin carotenoid levels or dietary antioxidant, either from male partners or female partners, contribute substantially to embryo development in IVF cycles for UI couples.

The second limitation is that we did not know exactly how the individual sperm cell or embryo was influenced by the skin carotenoid status and seminal TAC. We found that skin carotenoid status was correlated with day 3 good embryo rates. It indicated that the UI couples with high skin carotenoid status might obtain more good embryos and thereafter more good embryos available for transfer in IVF treatment. The two abortions in this study only occurred in the UI couples with low skin carotenoid status, and this resulted in significant difference of live birth rate compared with the UI couples with high skin carotenoid status. However, we need a large sample size and detail mechanism study to elucidate the effect of skin carotenoid status on individual spermatocyte and embryo.

In conclusion, the DGC method for sperm wash during IVF procedure increases the ROS levels but not oxidative DNA injury of spermatozoa. In contrast, the DGC method could remove, at least partially, the spermatozoa with DNA fragmentation. The local antioxidant status (seminal plasma TAC) is positively related to fertilization rates in IVF cycles for UI couples. The systemic carotenoid status of male partners of UI couples is closely relevant to embryo development in IVF cycles. Consequently, the skin carotenoid status of male partners of UI couples correlated with live birth outcome in IVF cycles due to the availability of good embryos for transfer. The effect of dietary antioxidant supplement on IVF cycles for both female and male partners of UI couples deserves further investigation.

## CONFLICT OF INTEREST

All the authors have no conflicts of interest to declare.

## ETHICAL APPROVAL

The study conforms to the Declaration of Helsinki, US, and/or European Medicines Agency Guidelines for human subjects. The approval of the Institution Review Boards of Chung Shan Medical University Hospital, Taichung, Taiwan (CS2‐17008), was obtained for all the study procedures. All the participating couples signed the informed consents.

## Supporting information

Data S1
